# Severe and mild drought cause distinct phylogenetically linked shifts in the blue grama (*Bouteloua gracilis*) rhizobiome

**DOI:** 10.3389/frmbi.2023.1310790

**Published:** 2024-01-11

**Authors:** Hannah M. Goemann, Danielle E. M. Ulrich, Brent M. Peyton, La Verne Gallegos-Graves, Rebecca C. Mueller

**Affiliations:** ^1^ Center for Biofilm Engineering, Montana State University, Bozeman, MT, United States; ^2^ Department of Microbiology and Cell Biology, Montana State University, Bozeman, MT, United States; ^3^ Department of Ecology, Montana State University, Bozeman, MT, United States; ^4^ Department of Chemical and Biological Engineering, Montana State University, Bozeman, MT, United States; ^5^ Thermal Biology Institute, Montana State University, Bozeman, MT, United States; ^6^ Bioscience Division, Los Alamos National Laboratory, Los Alamos, NM, United States; ^7^ United States Department of Agriculture (USDA) Agricultural Research Service, Western Regional Research Center, Albany, CA, United States

**Keywords:** soil microbiome, next-generation sequencing (NGS), drought, plant-microbe interaction, root exudate

## Abstract

Plants rely on a diverse rhizobiome to regulate nutrient acquisition and plant health. With increasing severity and frequency of droughts worldwide due to climate change, untangling the relationships between plants and their rhizobiomes is vital to maintaining agricultural productivity and protecting ecosystem diversity. While some plant physiological responses to drought are generally conserved, patterns of root exudation (release of small metabolites shown to influence microbes) and the consequential effects on the plant rhizobiome can differ widely across plant species under drought. To address this knowledge gap, we conducted a greenhouse study using blue grama (*Bouteloua gracilis*), a drought-tolerant C4 grass native to shortgrass prairie across North American plains, as a model organism to study the effect of increasing drought severity (ambient, mild drought, severe drought) on root exudation and the rhizobiome. Our previous results demonstrated physiological effects of increasing drought severity including an increase in belowground carbon allocation through root exudation and shifts in root exudate composition concurrent with the gradient of drought severity. This work is focused on the rhizobiome community structure using targeted sequencing and found that mild and severe drought resulted in unique shifts in the bacterial + archaeal and fungal communities relative to ambient, non-droughted controls. Specifically, using the change in relative abundance between ambient and drought conditions for each ZOTU as a surrogate for population-scale drought tolerance (e.g., as a response trait), we found that rhizobiome response to drought was non-randomly distributed across the phylogenies of both communities, suggesting that *Planctomycetota*, *Thermoproteota* (formerly *Thaumarchaeota*), and the *Glomeromycota* were the primary clades driving these changes. Correlation analyses indicated weak correlations between droughted community composition and a select few root exudate compounds previously implicated in plant drought responses including pyruvic acid, D-glucose, and myoinositol. This study demonstrates the variable impacts of drought severity on the composition of the blue grama rhizobiome and provides a platform for hypothesis generation for targeted functional studies of specific taxa involved in plant-microbe drought responses.

## Introduction

Drought is one of the most important abiotic factors limiting plant growth and development (de Vries et al., 2016; Berdugo et al., 2020). Increasing precipitation variability and rising temperatures due to climate change are predicted to increase drought intensity and the rate at which droughts develop (Mukherjee and Mishra, 2021). These changes will likely have dramatic consequences on the productivity and distribution of plant life in both agricultural and natural ecosystems. Understanding plant-microbe interactions in a wide variety of contexts is critical to making informed management decisions in agriculture and natural resource conservation in the face of global climate change.

The plant rhizosphere, the region of soil adjacent to plant roots that is directly influenced by plant growth, respiration, and nutrient exchange, is the epicenter of a complex web of plant-microbe interactions. The rhizosphere hosts a wide diversity of microorganisms including bacteria, archaea, and fungi collectively referred to as the rhizobiome that can improve plant access to nutrients and aid in plant survival under abiotic stress. Mutualistic fungi such as many arbuscular mycorrhizal fungi (AMF) can increase water exchange and nutrient mining to plants under drought stress in exchange for plant C inputs (Mathur et al., 2019). Bacteria including *Streptomyces* sp. (Abbasi et al., 2020; Li et al., 2020) and *Burkholderia* sp. (Naveed et al., 2014; Huang et al., 2017) have been utilized as plant inoculants to protect against drought stress. These and other drought-tolerant bacteria offer benefits to plants including the solubilization of mineral phosphates, nitrogen fixation, and production of plant growth-promoting hormones such as indole acetic acid (Passari et al., 2016; Dahal et al., 2017).

However, it remains difficult to discern broad patterns in rhizobiome community responses to drought stress due to variation in plant species, soil type, and climate. This is partly because we do not fully understand the influence of plant carbon inputs into the soil, namely as root exudates, on the rhizosphere microbiome. Root exudates are small molecules released by plant roots that frequently include sugars, amino acids, and organic acids (Bais et al., 2006). Many of these compounds have been found to have osmoprotectant or chemical signaling properties in various plant systems (Vries et al., 2019; Williams and de Vries, 2020). With recent advances in metabolomics technology, we are only beginning to understand the effects of changes in the quantity and composition of root exudates on rhizobiome community composition and function under increasing drought severity.

The goal of the present study was to examine how increasing drought severity influences the rhizobiome community diversity and composition of *B. gracilis*. Blue grama is a widespread, drought-tolerant C4 grass native to North America that accounts for 75-90% of net primary productivity of grasslands it inhabits and is a major contributor to belowground C storage (Costello, 1944). Blue grama is utilized for conservation, rangeland seeding, landscaping, reclamation, and erosion control and is also valuable for livestock and wildlife because it provides highly palatable forage year-round (Costello, 1944). The level of precipitation in the driest month of the year has historically been a strong influence on the distribution of blue grama across the central and western Great Plains from Canada to northern Mexico (Avendaño-González and Siqueiros-Delgado, 2021). The importance of the blue grama rhizobiome in mediating its drought tolerance is not well understood, although Ulrich et al. (2019) demonstrated that growing blue grama without its microbiome (grown in sterilized soil) resulted in less germination, less biomass, and lower drought tolerance compared to rhizobiome-intact plants. Prior results from this study indicated that increasing drought severity on blue grama grass (*Bouteloua gracilis*) resulted in strong shifts in root exudate composition with compounds such as sucrose, myo-inositol, pyruvic acid, and L-threonine primarily driving differences between drought severity treatments (Ulrich et al., 2022).

Here we further explored the above greenhouse experiment that subjected blue grama seedlings to three levels of drought severity by measuring the diversity and composition of the rhizosphere bacterial + archaeal and fungal communities using next generation amplicon sequencing. We tested two hypotheses: 1. Severe drought will have a stronger effect on the rhizobiome diversity and composition than mild drought and 2. drought-induced changes in root exudate composition would correlate to changes in rhizobiome composition. Understanding how increasing drought severity alters plant-soil feedbacks in drought-tolerant plants will provide important insights into plant-microbe mechanisms that may be applied to less drought-tolerant species (Mathur and Roy, 2021). Disentangling these relationships will also improve our abilities to model global nutrient dynamics and develop microbially enhanced food production and natural resource management solutions in the face of climate change.

## Methods

### Study site and experimental design

Blue grama was grown from commercial seed (Wind River Seed Co., Manderson, WY, USA) in a greenhouse (Bozeman, MT, USA) in small trays in a 50:50 mix of sand:soil collected from the top soil layer (0-10 cm) from a pinyon-juniper woodland near Bozeman, MT, which is the general ecosystem where blue grama naturally occurs (Coffin and Lauenroth, 1992; Goemann et al., 2024). This ensured microbes relevant to blue grama would be available (Naylor and Coleman-Derr, 2018). Seeds were planted on October 1, 2019. After 30 days of initial growth, 120 seedlings of similar size were selected and transplanted with the soil from the trays to larger pots (~0.75 L, 6.4 cm diameter, 25.4 cm height) with sterilized playground sand (Ulrich et al., 2019). Greenhouse conditions during the study (Oct-Jan 2019) consisted of a 16-hour photoperiod, daytime temperature of 23.8°C, nighttime temperature of 21.1°C, and average daytime PAR of 500 μmol m^2^ s^-1^. Plants were watered to field capacity and allowed to acclimate to greenhouse conditions for 30 days. Then, established plants were divided into three treatment groups: ambient, mild drought, and severe drought (n = 40 plants per treatment). The ambient treatment group received normal watering to field capacity. The mild drought treatment group was watered half as frequently to maintain 50% of field capacity. The severe drought treatment group was watered 25% as frequently to maintain 25% of field capacity. Treatments lasted 30 days. These drought severity levels were selected based on previous work where blue grama required 33 days of drought to desiccate when water was completely withheld (i.e. 100% reduction in water) (Ulrich et al., 2019). In this study, less severe drought treatment levels (50% and 75% *vs*. 100% reduction in water) were selected to induce plant stress response without killing the plants, allowing measurement of shifts in physiology and root exudate concentration and composition as a function of drought severity.

### Plant and soil measurements and collection

Plant physiology analysis is described in full in (Ulrich et al., 2022). Briefly, to determine how drought severity treatment influenced plant physiology, predawn leaf water potential, leaf gas exchange (photosynthesis, stomatal conductance) and root and shoot biomass were measured to determine root to shoot biomass ratios (root:shoot) in each treatment group before (T1) and after (T2) 30 days of treatment (December 19-January 18). Predawn leaf water potential was measured using a Scholander type pressure chamber (model 1505D, PMS Instruments, Corvallis, OR, USA). Gas exchange was measured using a portable photosynthesis instrument equipped with an infrared gas analyzer (LI-6800, Licor, Lincoln, NE USA) on 5 plants randomly selected from each treatment group. Plant physiology results demonstrating the effectiveness the drought treatments are listed in **Supplementary Figure S1**.

At time of harvest 50 mL each of rhizosphere and bulk soil fractions were collected separately, flash-frozen in an ethanol-dry ice bath and transferred to long-term storage at -80°C for microbial analysis. For rhizosphere collection a soil core was taken as close to the plant as possible directly adjacent to the root crown while the bulk soil core was collected close to the edge of the pot (~3 cm away). Root exudates were collected via vacuum filtration at the time of harvest and transferred to long-term storage at -80°C. Root exudate collection procedures followed the method of Phillips et al. (2008). Briefly, plants were removed from pots and roots were rinsed of soil in DI water and dipped in Gibco Antibiotic-Antimyotic solution (ThermoFisher Scientific CA, USA) to halt the microbial production of C compounds. This ensured that we collected exudates from the plant and not microbes. Plants were subsequently transplanted to filter flasks of 60-120 mesh glass beads (100 mL) that do not provide a C source but still provide mechanical pressure on the root system, resembling natural soil conditions. Plant roots were flushed with 100 mL of sterile water using a vacuum pump connected to the filter flasks, another 100 mL was added, and plants were allowed to release exudates over a 41-hour period. The remaining solution was filtered (0.22 µm) and collected for root exudate concentration and composition analyses. Root exudates were frozen and stored at −80°C until analysis. Exudate samples were submitted to the Environmental Molecular Science Laboratory (EMSL) for gas chromatography-mass spectrometry (GCMS) analysis and data preprocessing. GCMS methods are described in Ulrich et al. (2022).

### DNA extraction, amplification, and sequencing

To obtain high-resolution representation of the soil microbial community and its response to drought, we performed rRNA gene amplicon sequencing on all replicates of bulk and rhizosphere soil for all treatment and pretreatment (T1) samples (n=15 each for T1 rhizosphere and bulk soils, n=5 for post-drought (T2) rhizosphere and bulk samples across three treatments, total n=60). Genomic DNA was extracted from 0.5 g frozen soil samples using the Fast DNA SPIN Kit for soil (MP Biomedicals, Santa Ana, CA) according to the manufacturer’s instructions. We targeted the V4 region of the 16S rRNA gene using 515F (Parada et al., 2016) and r806b (GTGYCAGCMGCCGCGGTAA and GGACTACVSGGGTATCTAAT, respectively) from the Earth Microbiome Project (Thompson et al., 2017), and the D2 hypervariable region of the fungal large subunit (LSU) using LR22R (Mueller et al., 2016) and LR3. PCR reactions were carried out in 20 μL volumes containing 1 U Phusion high-fidelity polymerase (ThermoFisher Scientific, MA, USA), a final concentration of 1x Phusion HF reaction buffer, 200 μM dNTPs, 0.5 μM each primer, 1 μM BSA, 1 mM MgCl, sterile molecular grade water, and 5 μL (10 ng) genomic DNA as a template. PCR conditions consisted of initial denaturation at 98°C for 30s, followed by 22 cycles of 98°C for 30 s, 55°C for 30 s, 72°C for 45 s and a final extension at 72°C for five minutes. Samples were purified using AMPure XP (Beckman-Coulter, Brea CA, USA) magnetic beads and barcoding was conducted using the Illumina Nextera Indexing Kit D, with 10 cycles of indexing PCR in 20 μL volumes using the same concentrations as PCR1 with 10 μL template DNA. Samples were purified again with magnetic beads and PCR products were checked for quality and length in a 1% agarose gel and quantitated using the Quant-iT™ dsDNA Kit (Invitrogen, Carlsbad CA, USA) with a BioTek H2 plate reader. Individual samples for each community were combined at equimolar concentrations and the two libraries were pooled at 4 nM and loaded onto an in-house Illumina MiSeq (Illumina, San Diego CA, USA) and sequencing using the V3 600 cycle kit.

### Bioinformatics

Reads were merged, trimmed, and dereplicated with USEARCH, quality filtered with an ee-value of 1.0 and zero-radius operational taxonomic units (ZOTUs) were identified with UNOISE3 (v.11.0.667) (Edgar, 2010). A total of 4,668,982 16S and 4,872,815 LSU high quality reads were clustered into 15,541 and 6,893 ZOTUs respectively. Representative 16S and LSU ZOTUs were classified against their respective databases using a subset of the Genome Taxonomy Database (GTDB, Chaumeil et al., 2022) for 16S and the Ribosomal Database Project (RDP, Cole et al., 2014) v11 database for LSU using SINTAX (Edgar, 2016). All reads classified to chloroplast (16S), or animalia, protozoa, or viridiplantae (LSU) were removed prior to downstream analysis. For statistical analyses, datasets were randomly subsampled according to the sample with the lowest read number. To construct phylogenetic trees for environmental ZOTUs, we used reference phylogenies constructed using full length and near full length sequences of isolates and metagenome assemblies downloaded from GenBank, focusing on GTDB sequences for 16S and Assembling the Fungal Tree of Life sequences for LSU (Lutzoni et al., 2004). Reference sequences were aligned using mafft (v.7.250, Katoh and Standley, 2013), and a maximum likelihood tree was constructed with the general time reversible (GTR) + gamma model using RAxML (v7.2.7 Stamatakis, 2014). Environmental sequences were aligned to the reference using mafft with the –add flag and then mapped onto the reference tree using placer (v1.1.alpha19-0-g807fbf3, Matsen et al., 2010) with reference-aligned sequences. Trees were visualized and annotated using the interactive Tree of Life software (v6.8.1, Letunic and Bork, 2019).

### Statistics and data analysis

Community analyses were conducted using R software (v4.3.0, R, 2023) in RStudio (RStudio Team, 2020). Data management was handled primarily with the tidyverse package (Wickham et al., 2019). Community ordination and alpha diversity analyses were performed using the packages vegan (Oksanen et al., 2019), picante (Kembel et al., 2010) and phyloseq (McMurdie and Holmes, 2013). We used PERMANOVA (Anderson, 2017) to test for treatment, timepoint, and bulk *vs*. rhizosphere effects on community beta diversity computed using unweighted UniFrac (Lozupone and Knight, 2005). To explore the effect of time on community composition we conducted Wilcoxen Signed Rank Tests to test differences in relative abundances between T1 (pretreatment) and ambient samples at T2 (post-drought).

To examine whether ZOTU responses to drought were non-randomly distributed across the bacterial + archaeal and fungal phylogenies in the blue grama rhizosphere, we performed phylosignal analysis using response ratios as a response trait on T2 (post-drought) rhizosphere samples. Response ratios were computed by dividing the difference between the read counts in the treatment (severe or mild drought) and ambient conditions by the total read count for a ZOTU. Response ratios were permuted 999 times for each ZOTU and averaged across 5 replicate samples in each treatment. Phylosignal analyses were performed using phytools (Revell, 2012) and geiger (Pennell et al., 2014) for Pagel’s lambda and Blomberg’s K (Diniz-Filho et al., 2012). Packages phylobase (Hackathon et al., 2020) and adephylo (Jombart et al., 2010) were utilized for Abouheif’s C_mean_. One-way t-tests were then conducted across taxonomic levels to determine which clades had response ratios significantly different from 0. All t-tests were adjusted with Benjamini-Hochberg (BH) multiple comparisons corrections, and we report the adjusted p-values. To aid in visualization of the phylogenetic signal analysis, the response ratio for each ZOTU and for clades with significantly positive or negative clade-wide response ratios were mapped back to the phylogenetic trees using the color strip tree annotation template provided by the interactive Tree of Life software (Letunic and Bork, 2019).

Having identified several clades of bacteria, archaea, and fungi which had significant clade-wide response to severe drought, we performed network analysis to determine whether there were any detectable inter-kingdom (i.e. bacterial-fungal) associations in relative abundances between drought and ambient conditions. Sparcc networks were constructed and analyzed using the NetCoMi package (Peschel et al., 2021) in RStudio with specific parameters listed in the Supplementary Information. We utilized the differential network analysis module in the NetCoMi package to determine the effects of mild and severe drought on the top 50 of these potential associations. Differential network analysis uses permutation tests to assess whether associations between taxa are different between two user-defined treatment groups, in our case defined as mild drought *vs*. ambient and severe drought *vs*. ambient conditions (Peschel et al., 2021). All figures were edited for publishing quality in Adobe Illustrator 2023 27.5.0.

To calculate correlations between root exudation and community composition we selected root exudate compounds identified as driving the differences between treatment groups (ambient, mild drought, and severe drought) reported Ulrich et al. (2022). Correlation analysis was performed using the envfit function in the vegan package. Envfit correlation p-values were also BH-corrected for multiple comparisons.

## Results

### Drought effects on rhizobiome composition

**Figure 1:**
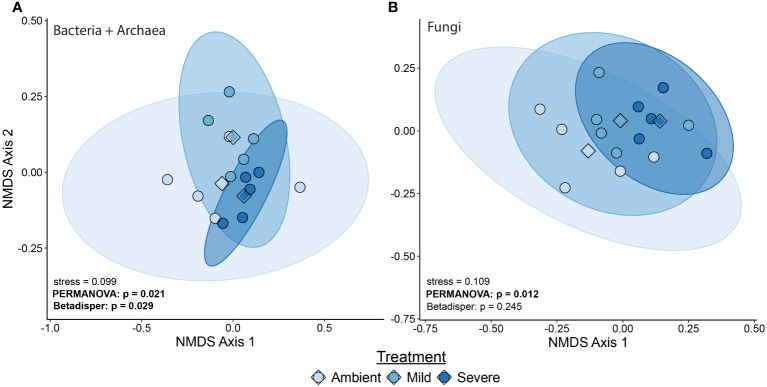
Beta diversity measured with unweighted Unifrac of bacterial/archaeal community **(A)** and fungal community **(B)** at T2. Points represent individual samples with a larger diamond representing the treatment centroid. Ellipses indicate the 95^th^ percentile area of community distribution. Legend shows symbol and ellipsis color.

**Figure 2:**
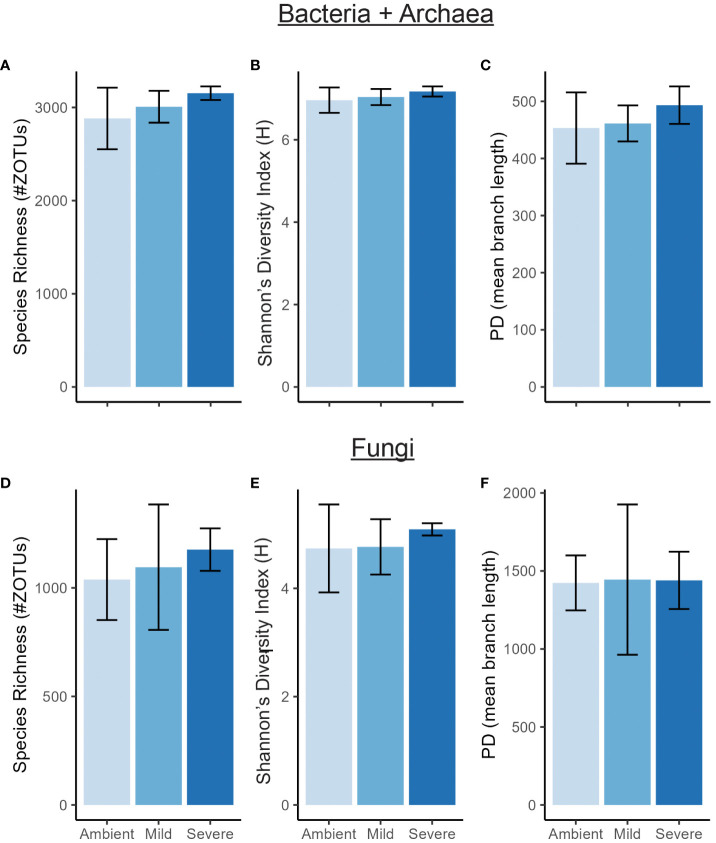
Alpha diversity of bacterial/archaeal community **(A–C)** and fungal community **(D–F)** measured by species richness, Shannon’s diversity index (H), and Faith’s Phylogenetic Diversity (PD) at T2. Error bars are ± SE.

We found severe drought resulted in significantly altered beta diversity of the bacterial + archaeal and fungal communities compared to the ambient treatment in T2 (Bacteria + archaea: F_2_ = 1.41, R^2^ = 0.0466, p = 0.01, Fungi: F_2_ = 1.34, R^2^ = 0.183, p = 0.025, **Figure 1**). In comparison, mild drought conditions had minimal effects on the rhizobiome beta diversity. We also detected decreased dispersion in the bacterial + archaeal community (F_2_ = 4.81, p = 0.029) in the severe drought condition compared to ambient. We did not find evidence for differences in alpha diversity between drought conditions (mild or severe) and ambient conditions or between mild and severe drought, in either the bacterial + archaeal or fungal communities across any of the three metrics tested: species richness, Shannon’s diversity, or Faith’s phylogenetic diversity (**Figure 2**; **Supplementary Table S1**), although we did detect a positive effect of time (T2 > T1) across all three metrics in the fungal community (**Supplementary Table S1**).

Beta diversity also differed between T1 and ambient conditions at T2 in both the bacterial + archaeal (F_2_ = 1.64, R^2^ = 0.0271, p = 0.007, **Table 1**) and fungal communities (F_2_ = 1.59, R^2^ = 0.0264, p = 0.059, **Table 1**), although the difference between T1 and T2 ambient conditions in both communities was slight. The differences between T1 and ambient-T2 in the bacteria + archaea were likely driven by the detection of *Armatimonadota* and *Nitrospirota* above 1% abundance only at T1, and detection of *Fibrobacterota* only in ambient-T2 samples (**Supplementary Figure S2A**). We did not detect any significant differences in relative abundances of fungal classes between T1 and ambient-T2 (**Supplementary Figure S2B**). We also did not detect differences between T1 and T2 in beta diversity between bulk and rhizosphere samples, or effects of any interactions between the combinations of soil compartment, time, and treatment (**Table 1**).

**Table 1:** Summary of beta diversity test statistics (PERMANOVA).

	Variable	Df	SumofSqs	R^2^	F	P-value
**Bacteria + Archaea**	Treatment (Drought)	2	0.471	0.0466	1.41	**0.01**
	Time	1	0.274	0.0271	1.64	**0.007**
	Microhabitat	1	0.173	0.0161	1.04	0.289
	Treatment : Time	2	0.383	0.0379	1.15	0.114
	Treatment : Microhabitat	2	0.333	0.0330	1.00	0.405
	Time : Microhabitat	1	0.160	0.0156	0.958	0.482
	Treatment : Time:Microhabitat	2	0.321	0.0318	0.965	0.553
	Beta dispersion	2	0.00336	NA	4.81	**0.029**
**Fungi**	Treatment (Drought)	2	0.370	0.1832	1.34	**0.025**
	Time	1	0.1706	0.02635	1.59	*0.059*
	Microhabitat	1	0.0803	0.01239	0.746	0.772
	Treatment : Time	2	0.2077	0.0321	0.965	0.477
	Treatment: Microhabitat	2	0.2412	0.0373	1.12	0.258
	Time: Microhabitat	1	0.078	0.0120	0.725	0.815
	Treatment : Time: Microhabitat	2	0.2633	0.0406	1.22	0.151
	Beta dispersion	2	0.001022	0.000511	0.766	0.486

Bold letters indicate significance at p < 0.05.

### Distribution of drought response across the rhizobiome phylogeny

Phylosignal analysis using response ratios as a response trait on the bacterial + archaeal and fungal trees indicated that changes in relative abundance between the mild drought and ambient treatments as well as the severe drought and ambient treatments were not randomly distributed across either tree (**Table 2**). In the bacterial + archaeal community, all three phylosignal tests (Pagel’s Lambda, Blomberg’s K, and Abouheif’s C_mean_) rejected the null hypothesis of random distribution of the response ratio trait. In the fungal community for both the mild drought-ambient and severe drought-ambient comparisons, Blomberg’s K test did not reject the null hypothesis but the other two tests were found to be significant.

**Table 2:** Summary of phylosignal test results.

Test	Mild Drought		Severe Drought	
	Stat	P-value	Stat	P-value
	**Bacteria + Archaea**			
Pagel’s Lambda	0.702	**2.21e-144**	0.701	**1.26e-144**
Blomberg’s K	0.0270	**0.001**	0.0179	**0.011**
Abouheif’s C_mean_	0.107	**0.001**	0.103	**0.001**
	**Fungi**			
Pagel’s Lambda	0.793	**1.51e-35**	0.808	**2.12e-51**
Blomberg’s K	0.0267	0.157	0.0287	0.134
Abouheif’s C_mean_	0.132	**0.001**	0.198	**0.001**

Bold lettering indicates significance at p < 0.05.

**Figure 3:**
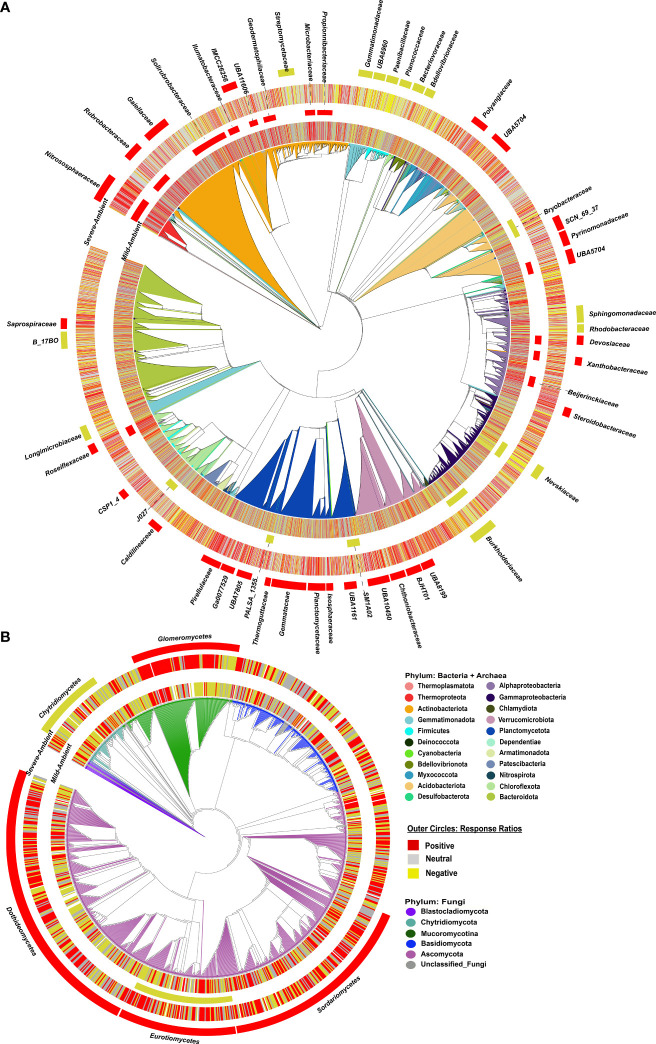
Phylogenetic trees of bacterial + archaeal **(A)** and fungal **(B)** communities, colored by phyla. The 1^st^ ring for each the Mild Drought-Ambient comparison (inner circles) and Severe Drought-Ambient comparison (outer circles) indicates positive (red), neutral (gray) or negative (yellow) response ratios at the ZOTU level, then families for the bacteria + archaea and classes for fungi with significantly positive (red) or negative (yellow) response ratios are indicated by red (positive) or yellow (negative) colored strips for both drought-ambient comparisons.

In the mild drought–ambient comparison, global analysis of response ratios detected one archaeal phylum, four bacterial phyla, and one fungal class which had clade-wide response ratios significantly different from zero (i.e., increased or decreased relative abundance in drought *vs*. ambient conditions). *Thermoproteota* (archaea also known as *Thaumarchaeota*), and bacterial *Chloroflexota* and *Actinobacteriota* had positive phylum-wide response ratios (greater relative abundance in mild drought *vs*. ambient conditions) while bacterial *Omnitrophota* and *Planctomycetota* had negative phylum-wide response ratios (**Supplementary Table S2**). The fungal class *Eurotiomycetes* within the *Ascomycota* was the only fungal clade detected and had a negative class-wide response ratio in the mild drought-ambient conditions comparison. At the family level for bacteria + archaea, we identified 15 families across the phylogeny with positive response ratios, and six with negative response ratios (**Figure 3A** inner circles, **Supplementary Table S2**). Most of the families with positive response ratios belonged to the *Actinobacteriota* phylum, including: *Rubrobacteraceae*, *Solirubrobacteraceae*, *Gaiellaceae*, *Illumatobacteraceae*, *Geodermatophilacaeae*, *Microbacteriaceae*, *Propionibacteriaceae*, and *UB11606* (order *Acidimicrobiales*). The other clades with positive response ratios included three families within the *Alphaproteobacteria* (*Devosiaceae*, *Xanthobacteraceae*, and *Beijerinckiacaeae*), *Roseiflexiaceae* (*Chloroflexota*), one *Acidobacteriota* family, *UBA5704 (Thermoanaerobaculia)*, and the only family present within the *Thermoproteota*, *Nitrososphaeraceae*. Negative family-wide responses included: *Bryobacteraceae* (*Acidobacteriota*), *Nevskiaceae* and *Burkholderiaceae* (*Gammaproteobacteria*), *SM1A02* and *PALSA_1355* (*Planctomycetota*) and *J027* (*Chloroflexota*).

In the severe drought-ambient comparison, we detected many more bacterial + archaeal clades with significantly positive or negative response ratios (11 phyla and 45 classes/families *vs*. 5 phyla and 22 classes/families in the mild drought-ambient comparison), with only a few overlapping between the two comparisons (**Figure 3A** outer circles, **Supplementary Table S2**). At the phylum level, the *Thermoproteota* and *Chloroflexota* maintained positive response ratios, while the *Planctomycetota* flipped from negative to positive. We also detected positive response ratios in the *Verrucomicrobiota* and *Acidobacteriota* which were not significant in the mild drought-response ratios. Likewise, the *Firmicutes*, *Gemmatimonadota*, and *Bdellovibrionota* were found to have negative response ratios only in the severe drought-ambient comparison. While the *Gemmatimonadota* have been associated with drought-resistance in some studies (Guo et al., 2023; Wang et al., 2022) there is evidence that members of this phylum are sensitive to dry-wet cycling (Qi et al., 2022) which the droughted samples experienced in this study. Although the *Actinobacteriota* did not have a significant phylum-wide response ratio in the severe drought-ambient comparison, we did detect positive response ratios in the *Rubrobacteraceae*, *Gaiellaceae*, and *IMCC26256* (order *Acidimicrobiia*) families within the *Actinobacteriota* as in the mild drought-ambient comparison. Unique to the severe drought-ambient comparison, actinobacteriotal family *Steptomycetaceae* had a negative response ratio. Within the *Proteobacteria* we observed positive response ratios in both drought treatments in the *Devosiaceae* and *Xanthobacteraceae*, and negative response ratios in both treatments in the *Burkholderiaceae* and *Nevskiaceae*. *Alphaproteobacteria* families *Sphingomonadaceae* and *Rhodobacteraceae* had negative response ratios unique to the severe drought-ambient comparison, and we observed a positive response ratio in the *Steroidobacteraceae* family in the severe drought-ambient comparison and a positive response in the *Beijerinckiaceae* family only in the mild drought-ambient comparison.

In the fungal community, severe drought also had a greater impact on response ratios than mild drought compared to ambient conditions. With the severe drought-ambient comparison we detected positive response ratios in the *Mucoromycotina* and *Ascomycota* phyla, and negative response ratio in the *Chytridiomycota.* We detected 4 classes which had positive response ratios including: *Glomeromycetes*, *Sordariomycetes*, *Dothideomycetes*, and the *Eurotiomycetes* (**Figure 3B**). The *Chytridiomycetes* were the only class with a clade-wide negative response ratio. Within the *Glomeromycetes* class there was a notable sub-clade, BLAST-identified as *Funneliformis* sp., which had negative response ratios although these did not influence the clade-wide positive response.

### Impact of drought severity on rhizobiome associations

**Figure 4:**
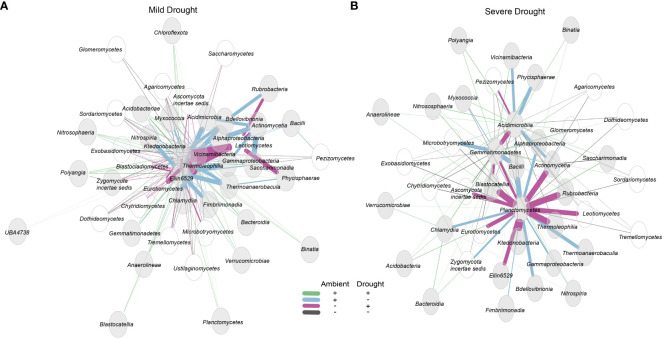
Sparcc differential network visualizing the top 50 associations of the total microbiome community at the class level under **(A)** mild drought vs. ambient conditions, and **(B)** severe drought vs. ambient conditions. Bacterial and archaeal nodes are gray while fungal nodes are unfilled. Line thickness indicates strength of association. All associations are significant with BH-adjusted p < 0.05.

To determine the effects of drought severity on community associations, we performed network analyses using differential networks of mild drought-ambient and severe drought-ambient communities. In the mild drought-ambient differential network, we identified a number of bacterial-fungal associations affected by drought (**Figure 4A**). *Acidobacteriotal* class *Vicinamibacteria*, *Actinobacteriota* class *Thermoleophilia*, and *Chloroflexota* classes *Ktdedonobacteria* and *Ellin6529* were the most connected classes in the network. The fungal class *Leotiomycetes* appeared to have several associations with bacterial classes that were negative under ambient conditions but positive under mild drought conditions (pink lines) including associations with *Ktedonobacteria*, *Thermoleophilia*, *Actinomycetia*, *Rubrobacteria*, and *Phycisphaerae.* In contrast, bacterial class *Actinomycetia* had several associations that were positive under ambient conditions but negative under mild drought (blue lines) including associations with *Acidimicrobia*, *Alphaproteobacteria*, *Gammaproteobacteria*, and *Saccharimonadia*. Some classes such as *Binatia*, *Planctomycetes*, and *Blastocatellia* had only positive associations with other taxa in both ambient conditions (green lines). Several fungal classes had primarily negative associations regardless of treatment group (black lines) including the *Glomeromycetes*, *Pezizomycetes*, *Dothideomycetes*, and *Sordariomycetes*.

In the severe drought-ambient differential network, *Planctomycetes* was the most connected class and appeared to have strong tradeoffs of positive associations in the ambient *vs*. severe drought communities. *Planctomycetes* associated negatively with ten classes in the ambient condition that were positive associations in the severe drought condition (purple lines) including *Actinomycetia*, *Blastocatellia*, *Ellin* 6529, *Gemmatimonadetes*, *Ktedonobacteria, Rubrobacteria*, Thermoleophilia, and fungal classes *Ascomycota incertae sedis, Eurotiomycetes*, and *Leotiomycetes*. In contrast, *Planctomycetes* were negatively associated with a different set of seven classes in the severe drought condition that were positive associations in ambient condition. These included *Alphaproteobacteria*, *Bacilli*, *Bdellovibrionata*, *Chlamydiia*, *Gammaproteobacteria*, *Thermoanaerobaculia*, and *Zygomycota incertae sedis Nitrososphaeraceae* was always positively associated with *Planctomycetes* while the majority of the fungal classes including the *Tremellomycetes*, *Agaricomycetes*, *Microbotryomycetes*, *Chytridiomycetes*, *Exobasidiomycetes*, and *Dothidiomycetes* were always negatively associated with *Planctomycetes* regardless of treatment.

### Connections to plant physiology & root exudation

Previous results focusing on changes in blue grama root exudation in response to increasing drought severity found significantly increased total organic carbon in root exudate extracts under severe drought compared to ambient conditions (Ulrich et al., 2022). There were ten root exudate compounds driving the greatest differences in exudate composition across the three treatments (ambient, mild drought and severe drought). These compounds were sucrose, D-arabinose, pyruvic acid, D-mannose, sedoheptulose anhydride monohydrate, tagatose, D-glucose, L-threonine, and 4-guanidinobutyric acid (Ulrich et al., 2022). Here we detected weak evidence for a correlation between the composition of these root exudates across samples with the rhizosphere bacterial + archaeal (Mantel R^2^ = 0.299, p = 0.084) and fungal (Mantel R^2^ = 0.289, p = 0.087) community compositions (beta diversity). In the bacterial + archaeal community, the best possible model correlating root exudates to community composition included only myoinositol and D-arabinose (Mantel R^2^ = 0.269, p=0.081). For the fungal community, the best possible model included pyruvic acid, D-mannose, tagatose, and D-glucose (Mantel R^2^ = 0.296, p = 0.083). Pearson correlations between taxa with significant clade-wide response ratios (i.e., those indicated by **Figure 3**) and root exudates were also conducted but no significant correlations were detected.

## Discussion

### Severe drought had stronger effects on the blue grama rhizobiome than mild drought

In support of our first hypothesis, severe drought had a greater impact on the blue grama rhizobiome composition than mild drought compared to ambient, non-droughted controls. Shifts in overall composition of the bacterial + archaeal and fungal communities were detected, and a decrease in dispersion (e.g., reduced beta diversity) among bacterial + archaeal community members was observed during severe drought conditions compared to the ambient treatment (**Figure 1**). In contrast, mild drought did not induce a significant shift in beta diversity among either the bacteria + archaea or fungi (**Figure 1**). The greater impact of severe drought on the soil rhizobiome compared to mild (or moderate) drought has been hypothesized in modeling efforts such as demonstrated by Wang and Allison (2021). Simmons et al. (2020) also demonstrated that severe drought had a greater impact than moderate drought on rhizosphere bacterial community composition across multiple species of millet.

The lack of drought treatment effects (either mild or severe) on alpha diversity in either the bacterial + archaeal or fungal communities (**Figure 2**) indicated that the changes in community composition were primarily due to shifts in relative abundances of existing members. Importantly, greenhouse studies limit dispersal which can have significant effects on community diversity under abiotic stress (Evans et al., 2020). However, the controlled conditions of greenhouse studies allow for treatment effect isolation and deliberately limiting dispersal effects improves the ability to detect shifts more directly in existing community membership. Phylogenetic signal is considered a useful metric for studying the effects of drought on the soil microbiome as many clades at different taxonomic levels tend to respond roughly in unison (Naylor et al., 2017; Koyama et al., 2018; Xu et al., 2018). Accordingly, phylogenetic signal in comparisons of both mild drought-ambient and severe drought-ambient rhizobiomes were detected using response ratios of the differences in relative abundances of taxa between treatment groups as a response trait (**Figure 3**).

Several clades of microorganisms have been found to be resilient to drought, including the *Actinobacteriota*, *Firmicutes*, *Mucoromycotina* (which includes the *Glomeromycetes*), and various subclades among the *Ascomycota* (Xu et al., 2018; Santos-Medellín et al., 2021). Consistently, we observed positive clade-wide responses (i.e. increased relative abundance in severe drought *vs*. ambient conditions) in all of these phyla with the exception of the *Firmicutes*, which in our study exhibited a clade-wide negative response in severe drought (i.e. decreased relative abundance *vs*. ambient conditions) but not mild drought. Overall, severe drought resulted in a greater number of significant response ratio changes (either positive or negative) across the bacterial + archaeal and fungal phylogenies than mild drought. For the fungal community in particular, the lack of significant response under mild drought conditions may reflect the greater water stress tolerance amongst mycelial community members (Santos-Medellín et al., 2021). Plant-microbe interactions are often the most beneficial when they help mediate access to the most limiting nutrient in an environment, such as water during drought stress (Ruth et al., 2011). Due to the lack of strong compositional effects in mild drought conditions, we hypothesize that blue grama may be able to self-regulate and tolerate mild drought. However, the greater investment in root exudation observed under severe drought stress (Ulrich et al., 2022) may be a mechanism to stimulate microbial mutualisms (i.e. the positive response ratios across the mycorrhizal *Glomeromycetes*) to help alleviate severe drought stress (Bécard and Piché, 1989; Vries et al., 2019).

Within the *Actinobacteriota*, more positive response ratios were detected in the mild drought than severe drought compared to ambient conditions, although some families such as *Rubrobacter*, which are known to be highly tolerant to desiccation (Naylor et al., 2017; Tóth et al., 2017), had positive response ratios in both drought severity treatments. Interestingly, while the actinobacteriotal genus *Streptomyces* has been found to alleviate drought stress in multiple agricultural plant species including tomatoes (Abbasi et al., 2020), maize (Warrad et al., 2020), and wheat (Li et al., 2020), here we observed a decrease in relative abundance of *Streptomycetaceae* in response to severe drought compared to ambient. Likewise, while we detected decreased relative abundances of *Burkholderiaceae (Alphaproteobacteria)* in both mild and severe drought compared to ambient, *Burkholderia* sp. have been used as plant inoculants to successfully protect against drought stress in *Arabidopsis*, maize, wheat, tomato, and bell peppers (Naveed et al., 2014; Tallapragada et al., 2016; Huang et al., 2017). Importantly, inoculants are most commonly applied to annual, domesticated crops with the exception of some trees (Umashankar et al., 2012; Bizos et al., 2020). Wild, perennial plant species such as blue grama typically have greater genetic diversity and often greater natural drought tolerance than cultivated species (Budak et al., 2013) which likely results in unique drought response patterns in the rhizobiome. The results presented here may also serve to broaden the known range of drought response patterns across plant species.

While the majority of the *Mucoromycotina* had positive response ratios in the severe drought-ambient comparison, we observed a distinct sub-group of *Funneliformis* sp. within the *Glomeromycetes* which had negative response ratios. This negative response is surprising as *Funneliformis* sp. are arbuscular mycorrhizal fungi which have been utilized as inoculants for several plants including thyme, soybeans and groundcherries to improve plant growth during water stress (Reyes et al., 2019; Amani Machiani et al., 2021). However, recent studies of bacterial colonization of mycorrhizal spores (Schrey et al., 2012; Lasudee et al., 2018) have identified *Streptomyces* sp. as among the most common endophytic mycorrhizal colonizers, and of *Funneliformis* sp. specifically. A tight association between *Funneliformis* sp. and *Streptomyces* sp. may explain the negative response ratio we observed in the *Streptomycetaceae* in the severe drought-ambient comparison. These results are also further evidence that drought-tolerant blue grama may not rely on the same drought response mechanisms that have been observed in other plant species.

### Insights on inter-kingdom associations via network analysis

Network analysis enables the identification of co-occurrence patterns between microbial taxa which may provide clues to shared niche spaces, nutrient acquisition strategies, or even direct symbioses (Barberán et al., 2012). These analyses provide an important foundation for hypothesis generation through which future experiments can be designed to determine the extent and conditionality of associations between taxa. Here we observed several distinct network patterns between mild drought-ambient and severe drought-ambient networks (**Figure 4**).

In the severe drought-ambient differential network the *Planctomycetes* was the bacterial class most highly connected to other bacterial and fungal classes. There were ten negative associations with *Planctomycetes* in ambient conditions which were positive associations in severe drought conditions, seven associations which were positive in ambient conditions but negative in severe drought conditions, three that were always positive regardless of treatment, and seven which were always negative regardless of treatment conditions. *Planctomycetota* are among the most commonly detected clades in aquatic and terrestrial ecosystems (Delgado-Baquerizo et al., 2018; Wiegand et al., 2018), but due to their low cultivability, little is known about the extent of their metabolic capabilities and influence on nutrient cycling. Wang et al. (2015) reported the ability of *Planctomycetota* species to degrade complex heteropolysaccharides in soil. Elevated or maintained relative abundance of *Planctomycetota* in response to drought has also been observed in several studies (Sheik et al., 2011; Dai et al., 2019). In addition, some *Planctomycetota* have been found to display antifungal properties. Graça et al. (2016) explored 40 *Planctomycetota* isolates to identify bioactive molecules and found that 43% of their study group contained genetic markers for antifungal activity which aligns with our finding that many fungal classes were negatively associated with *Planctomycetes* regardless of treatment condition. Although this apparent relationship was not influenced by drought and was not detected at all in mild drought conditions, it is additional evidence of possible functional attributes of a largely uncharacterized clade of microorganisms that warrants further investigation.

The mild drought-ambient differential network was largely unique from the severe drought-ambient network. Here, *Planctomycetes* only exhibited positive associations with the most connected taxa. These most connected taxa included *Thermoleophilia* (*Actinobacteriota*), *Ktedonobacteria* (*Chloroflexota*), and *Vicinamibacteria* (*Acidobacteriota*). *Ktedonobacteria* have been identified as a keystone specialist group in tundra ecosystems with positive correlations to lower moisture content (Wong et al., 2023) and while much of their metabolic capability remains undetermined, Zheng et al. (2019) observed strong functionality related to plant decomposition through cellulolytic activity. Although *Vicinamibacteria* were not detected in the mild drought-ambient comparison of our response ratio analysis, closely related *Bryobacteraceae* exhibited a negative response, and family *SCN_69_37* of the *Vicinamibacteria* did have a positive response ratio in the severe drought-ambient condition. *Vicinamibacteria* have also previously been reported as highly drought tolerant (Huber et al., 2022) although their mechanism of survival is not yet understood.

The differential networks were further utilized to investigate possible inter-kingdom associations of interest. We hypothesized that the observed clade-wide positive response observed in the response ratios of the *Thermoproteota* (ammonia-oxidizing archaea) under severe drought may be linked to the concurrent increase in *Glomeromycetes* (arbuscular mycorrhizal fungi) relative abundance via turnover of organic N-rich root exudates such as urea, uracil and various amino acids that were detected under the severe drought conditions (Ulrich et al., 2022). Arbuscular mycorrhizal fungi (AMF) are well known plant growth promoters and can help alleviate drought stress through hyphal mining for water and nutrients in the bulk soil adjacent to plant roots (Wu et al., 2013; Mathur et al., 2019). However, due to low saprotrophic ability, AMF proliferation is highly reliant on other soil microbes to degrade organic compounds and release available nitrogen (Bukovská et al., 2016). Despite exhibiting strong phyla-wide positive response ratios in severe drought conditions, network analysis did not detect any direct associations between *Thermoproteota* and *Mucoromycotina*. Importantly, although sparcc network statistics are compositionally aware, users are required to manually set thresholds for taxa inclusion that may bias networks towards more highly abundant taxa (MatChado et al., 2021). Members of *Thermoproteota* were among the most abundant ZOTUs so associations with the relatively rarer *Mucoromycotina* may exist but were not detected in the sparcc analysis. Future work would also benefit from the inclusion of non-plant controls to disentangle plant-specific microbiome responses distinct from responses of the soil microbiome alone to drought (Bandopadhyay et al., 2023).

### Lack of difference in rhizosphere *vs*. bulk samples suggests a wider scope of the rhizosphere zone of influence

Differentiating rhizosphere from bulk soil is common practice in soil microbial ecology studies to determine the influence of plants on the soil microbiome, and differences in community composition between rhizosphere and bulk soils have been well-documented (Castellano-Hinojosa et al., 2021; Ling et al., 2022). However, here we did not detect any significant differences between rhizosphere and bulk soil samples. We hypothesize that the small diameter of our pots (6.4 cm) did not allow for sufficient coring distance from plant roots to make this distinction as the roots largely filled the available space. This finding challenges a common working definition of rhizosphere soil as that which is specifically attached to roots (Chaparro et al., 2014; Zhu et al., 2020; Shami et al., 2022). Kuzyakov and Razavi (2019) reviewed the temporal dynamics and spatial stationarity of plant rhizospheres and found that the rhizosphere generally extends 0.5-4 mm from plant roots. Due to the 4-dimensional (3D-space + 1D-time), fibrous, high-density and high turnover rate of root structure of most grasses including *B. gracilis* (Ravenek et al., 2016), it is very possible that the vast majority of the soil within our pots were within this zone of influence. In a recent global meta-analysis of rhizosphere *vs*. bulk soil microbiomes, Ling et al. (2022) also found minimal differences in alpha diversity between bulk and rhizosphere soils in grasslands. Since plant species, soil type and texture, nutrient availability and microbiome activity all influence plant-microbe interactions (Jiang et al., 2017; Ling et al., 2022), it follows that the size of the zone of influence of the rhizosphere would be similarly variable.

### Root exudates weakly correlate to microbiome composition

In partial support of our second hypothesis, potential exudate compounds of interest which were primarily sugars and organic acids weakly correlated with the microbiome composition metrics. One possible compound of interest is pyruvic acid which is typically in high abundance for normal functioning photosynthesis, glycolysis, and the TCA cycle. Maurer et al. (2021) found high levels of pyruvate and amino acid exudation that had stimulatory effects on denitrification enzyme activity. Interestingly, while some compounds such as urea, arbutin, L-proline, and D-gluconic acid were only detected in the mild and severe drought treatment root exudates (Ulrich et al., 2022), no correlations between these compounds and shifts in the microbial community composition were detected here. As suggested by Bandopadhyay et al. (2023), perennial grass species may not be as reliant on stress signaling to microbes via root exudation due to their relatively greater investment in belowground root architecture compared to many annual plant species. Another possibility, as suggested by Karlowsky et al. (2018), is that root exudation during drought may be more important for the reinitiation of soil microbial activity following rewetting of the system. The authors suggest that further studies incorporating recovery from drought are needed to test this hypothesis across different soils, plants, and environments. In addition, utilizing metatranscriptomics to measure differential expression of metabolic pathways in the rhizobiome under drought will also be an important step in linking plant-microbe responses to drought. This would bypass the limitations of DNA-based analyses which cannot distinguish the contribution of active microbes to ecosystem processes (Carini et al., 2016). Metatranscriptomics would also provide evidence for potential utilization of root exudates. Experiments could then be designed to explore specific relationships between active microbial taxa and plant physiology.

## Conclusion

Here we show that the blue grama rhizobiome underwent significant community shifts as a result of severe drought and was only marginally affected by mild drought. We detected phylogenetically linked tradeoffs in community composition as a result of severe drought conditions including increased relative abundances of arbuscular mycorrhizae (*Glomeromycetes*), ammonia oxidizing archaea (*Thermoproteota*), and *Planctomycetota*. The bacterial class *Planctomycetes* was also the most highly connected to other bacterial and fungal classes in severe drought conditions, suggesting possible importance in the blue grama drought response. We also observed negative response ratios in several typically drought-tolerant bacterial clades including the *Firmicutes, Gemmatimonadetes*, and actinobacterial *Streptomyces* that challenge the perception of their conserved response to drought across plant rhizobiomes and soil ecosystems. Although we detected weak evidence for correlations between root exudate composition and community beta diversity, these analyses provide a platform for future exploration of similar multi-omic datasets. Altogether this study provides an important step towards understanding plant-microbe feedbacks under drought conditions and provides a foundation for future targeted investigations of the potential protective effects of root exudates and the rhizobiome against drought stress.

## Data availability statement

The datasets presented in this study can be found in online repositories. The names of the repository/repositories and accession number(s) can be found below: https://www.ncbi.nlm.nih.gov/, PRJNA1006788.

## Author contributions

HG: Data curation, Formal analysis, Methodology, Resources, Software, Validation, Visualization, Writing – original draft, Writing – review & editing, Investigation. DU: Supervision, Validation, Visualization, Writing – review & editing, Project administration, Resources, Conceptualization, Funding acquisition, Investigation, Methodology. BP: Project administration, Resources, Supervision, Validation, Visualization, Writing – review & editing. LG: Conceptualization, Funding acquisition, Resources, Writing – review & editing, Methodology. RM: Methodology, Writing – review & editing, Investigation, Supervision, Validation, Visualization.
