# Supplementary material for: Severe and mild drought cause distinct phylogenetically linked shifts in the blue grama (Bouteloua gracilis) rhizobiome
Source: Front Microbiomes. 2024 Jan 11;2:1310790. doi: 10.3389/frmbi.2023.1310790 (PMC12993653; doi:10.3389/frmbi.2023.1310790)
Supplement: Supplementary file 1 [file DataSheet_1.docx]

# Title:

Severe and mild drought cause distinct phylogenetically linked shifts in the blue grama (*Bouteloua gracilis*) rhizobiome

Hannah M. Goemann^1,2^, Danielle E. M. Ulrich^3^, Brent M. Peyton^1,4,5^, La Verne Gallegos-Graves^6^, Rebecca C. Mueller^1,7*^

1. Center for Biofilm Engineering, Montana State University, Bozeman, MT USA

2. Department of Microbiology and Cell Biology, Montana State University, Bozeman, MT USA

3. Department of Ecology, Montana State University, Bozeman, MT USA

4. Department of Chemical and Biological Engineering, Montana State University, Bozeman, MT USA

5. Thermal Biology Institute, Montana State University, Bozeman, MT USA

6. Bioscience Division, Los Alamos National Laboratory, Los Alamos, NM USA

7. USDA Agricultural Research Service, Western Regional Research Center, Albany CA

* Correspondence:

**Rebecca C. Mueller**

[rebecca.mueller@usda.gov](mailto:bpeyton@montana.edu)

# Supplemental Information

**Table S1.** Summary of P-values from linear mixed effects models describing the effects of treatment, day, microhabitat (bulk vs. rhizosphere) and their interactions on microbiome alpha diversity.

|  | **Predictor** | **Richness (#Zotus)** | **Shannon’s Diversity (H)** | **Faith’s Phylogenetic Diversity (mbl)** |
| --- | --- | --- | --- | --- |
| Bacteria+  Archaea | Treatment | 0.284 | 0.186 | 0.410 |
|  | Microhabitation | 0.884 | 0.583 | 0.393 |
|  | Time | 0.158 | 0.583 | 0.123 |
|  | Treatment*Time | 0.608 | 0.490 | 0.699 |
|  | Treatment*Microhabitat | 0.197 | 0.377 | 0.120 |
|  | Time*Microhabitat | 0.326 | 0.244 | 0.273 |
|  | Treatment*Time*Microhabitat | 0.232 | 0.356 | 0.175 |
| Fungi | Treatment | 0.503 | 0.613 | 0.642 |
|  | Microhabitation | 0.761 | 0.983 | 0.421 |
|  | Time | **< 0.001** | **0.002** | **< 0.001** |
|  | Treatment*Time | 0.187 | 0.364 | 0.403 |
|  | Treatment*Microhabitat | 0.191 | *0.0597* | **0.0356** |
|  | Time*Microhabitat | 0.299 | *0.0856* | 0.157 |
|  | Treatment*Time*Microhabitat | 0.769 | 0.145 | 0.674 |

**Table S2**. Summary of T-Test results for clade-wide response ratios

|  | **Comparison** | **Taxonomy** | **Stat** | **p.adj** |
| --- | --- | --- | --- | --- |
| **Bacteria + Archaea** | Mild-Ambient | **Phylum** |  |  |
|  |  | *Omnitrophota* | -0.485 | 0.0434 |
|  |  | *Planctomycetota* | -0.0371 | 0.0485 |
|  |  | *Chloroflexota* | 0.0581 | 0.0173 |
|  |  | *Actinobacteriota* | 0.12 | < 0.001 |
|  |  | *Thermoproteota* | 0.246 | < 0.001 |
|  |  | **Family** |  |  |
|  |  | *J027* | -0.359 | 0.0162 |
|  |  | *Burkholderiaceae* | -0.106 | 0.0405 |
|  |  | *SM1A02* | -0.29 | 0.0461 |
|  |  | *PALSA_1355* | -0.318 | 0.0167 |
|  |  | *Bryobacteraceae* | -0.176 | 0.00451 |
|  |  | *Nevskiaceae* | -0.667 | < 0.001 |
|  |  | *Beijerinckiaceae* | 0.281 | < 0.001 |
|  |  | *Devosiaceae* | 0.237 | 0.0196 |
|  |  | *UBA5704* | 0.392 | 0.0161 |
|  |  | *Propionibacteriaceae* | 0.282 | < 0.001 |
|  |  | *Geodermatophilaceae* | 0.268 | < 0.001 |
|  |  | *Microbacteriaceae* | 0.143 | 0.0196 |
|  |  | *UBA11606* | 0.234 | 0.0461 |
|  |  | *Ilumatobacteraceae* | 0.158 | 0.0178 |
|  |  | *Gaiellaceae* | 0.221 | < 0.001 |
|  |  | *Solibrubrobacteraceae* | 0.112 | < 0.001 |
|  |  | *Rubrobacteraeceae* | 0.189 | < 0.001 |
|  |  | *Nitrososphaeraceae* | 0.246 | < 0.001 |
|  |  | *Xanthobacteraceae* | 0.109 | < 0.001 |
|  |  | *Roseiflexaceae* | 0.294 | 0.0001 |
|  |  | *Ktedonobacteraceae* | 0.145 | 0.027 |
|  | Severe-Ambient | **Phylum** |  |  |
|  |  | *Firmicutes* | -0.33 | < 0.001 |
|  |  | *Gemmatimonadota* | -0.153 | < 0.001 |
|  |  | *Bdellovirionota* | -0.144 | 0.00503 |
|  |  | *Acidobacteriota* | 0.054 | 0.00115 |
|  |  | *Chloroflexota* | 0.0971 | < 0.001 |
|  |  | *Verrucomicrobiota* | 0.144 | < 0.001 |
|  |  | *Planctomycetota* | 0.153 | < 0.001 |
|  |  | *Thermoproteota* | 0.299 | < 0.001 |
|  |  | **Family** |  |  |
|  |  | *Nevskiaceae* | -0.435 | < 0.001 |
|  |  | *Rhodobacteraceae* | -0.359 | 0.0378 |
|  |  | *Paenibacillaceae* | -0.34 | < 0.001 |
|  |  | *UBA 6960* | -0.337 | 0.0163 |
|  |  | *Bedellovibrionaceae* | -0.331 | 0.00727 |
|  |  | *Longimicrobiaceae* | -0.315 | 0.00669 |
|  |  | *B_17BO* | -0.22 | 0.0101 |
|  |  | *Streptomycetaceae* | -0.189 | 0.00458 |
|  |  | *Bacteriovoraceae* | -0.166 | 0.0456 |
|  |  | *Planococcaceae* | -0.155 | 0.0199 |
|  |  | *Gemmatimonadaceae* | -0.151 | < 0.001 |
|  |  | *Sphingomonadaceae* | -0.127 | 0.0198 |
|  |  | *Burkholderiaceae* | -0.127 | 0.0031 |
|  |  | *Pyrinomonadaceae* | 0.0838 | 0.00919 |
|  |  | *Gemmataceae* | 0.0934 | 0.00458 |
|  |  | *Rubrobacteraceae* | 0.0999 | 0.00715 |
|  |  | *UBA1161* | 0.106 | 0.0137 |
|  |  | *UBA10450* | 0.11 | 0.0378 |
|  |  | *Gaiellaceae* | 0.127 | < 0.001 |
|  |  | *Polyangiaceae* | 0.146 | 0.0163 |
|  |  | *Chthoniobacteraceae* | 0.149 | 0.0163 |
|  |  | *SCN_69_37* | 0.156 | 0.0455 |
|  |  | *Caldilineaceae* | 0.171 | 0.0125 |
|  |  | *Steroidobacteraceae* | 0.173 | 0.0121 |
|  |  | *Saprospiraceae* | 0.173 | 0.0455 |
|  |  | *UBA7805* | 0.174 | 0.0353 |
|  |  | *CSP1_4* | 0.186 | 0.00155 |
|  |  | *Planctomycetaceae* | 0.191 | 0.0125 |
|  |  | *Xanthobacteraceae* | 0.191 | < 0.001 |
|  |  | *Pirellulaceae* | 0.205 | < 0.001 |
|  |  | *IMCC26256* | 0.211 | 0.00727 |
|  |  | *BJHT01* | 0.211 | 0.0112 |
|  |  | *UBA8199* | 0.211 | 0.0188 |
|  |  | *Devosiaceae* | 0.217 | 0.0455 |
|  |  | *Ga0077529* | 0.228 | < 0.001 |
|  |  | *Roseiflexaceae* | 0.247 | 0.00386 |
|  |  | *Isosphaeraceae* | 0.305 | 0.00423 |
|  |  | *Nitrososphaeraceae* | 0.312 | < 0.001 |
|  |  | *Thermoguttaceae* | 0.361 | < 0.001 |
|  |  | *UBA5704* | 0.411 | 0.000472 |
| **Fungi** | Mild-Ambient | **Class** |  |  |
|  |  | *Eurotiomycetes* | -0.17 | 0.009 |
|  | Severe Ambient | **Phylum** |  |  |
|  |  | *Chytridiomycetes* | -0.314 | 0.00571 |
|  |  | *Ascomycota* | 0.191 | < 0.001 |
|  |  | *Mucoromycotina* | 0.512 | < 0.001 |
|  |  | **Class** |  |  |
|  |  | *Chytridiomycetes* | -0.33 | 0.00655 |
|  |  | *Dothideomycetes* | 0.179 | 0.0019 |
|  |  | *Eurotiomycetes* | 0.188 | 0.000151 |
|  |  | *Sordariomycetes* | 0.222 | < 0.001 |
|  |  | *Glomeromycets* | 0.512 | < 0.001 |


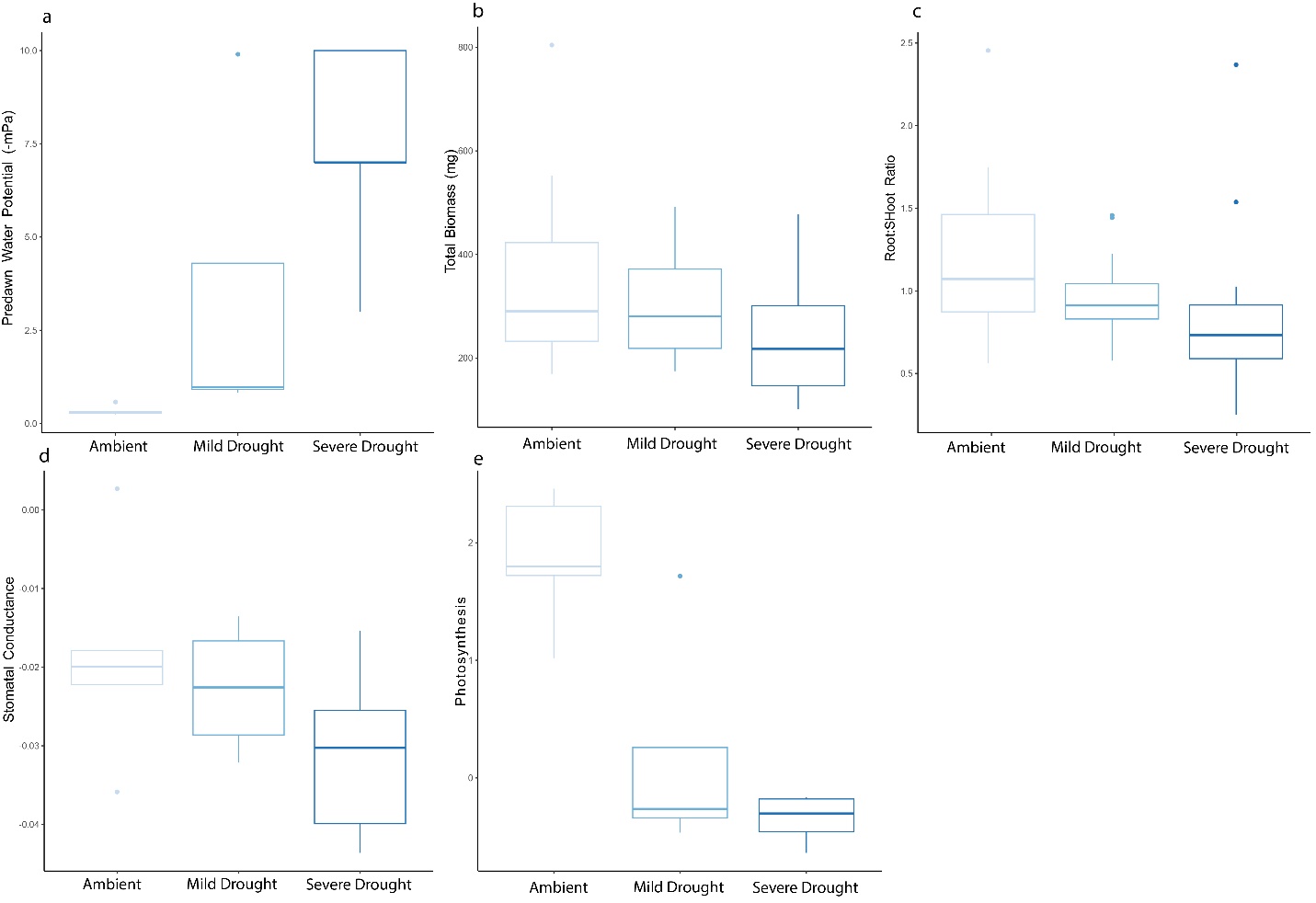


Figure S1. Plant physiology at T2 in response to increasing drought severity.


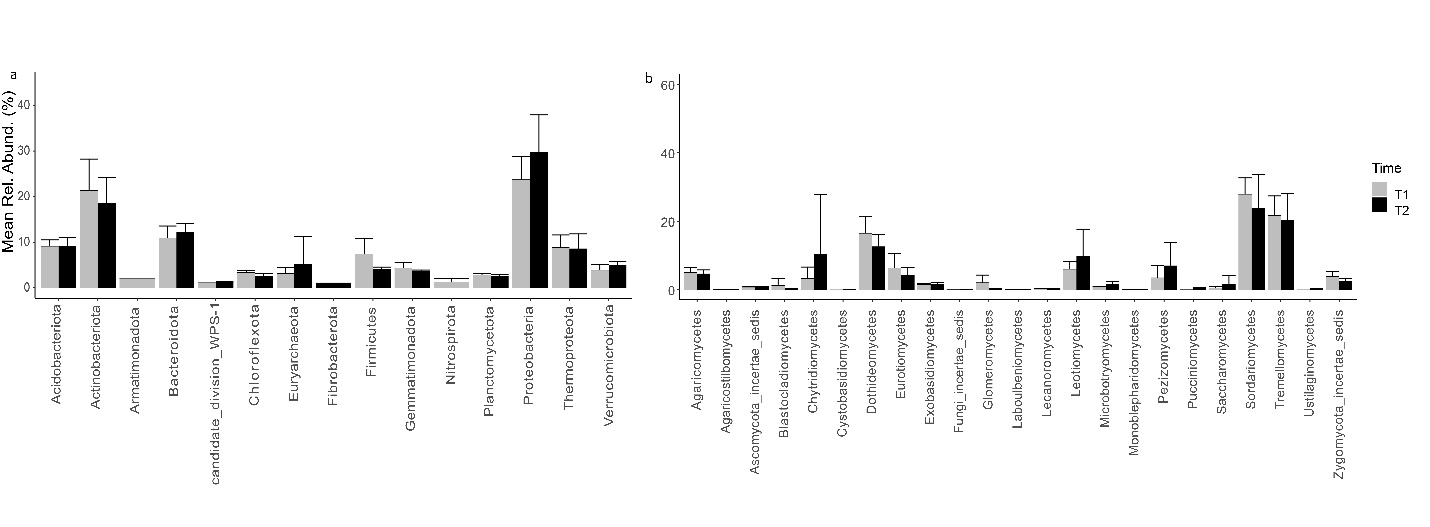


Figure S2. Relative abundances of (a) bacteria + archaeal phyla, and (b) fungal classes at T1 vs. ambient T2. * indicates p < 0.05 based on BH-corrected two-way Wilcoxen tests. Error bars are 95% CI.

**NetCoMi Parameters:**

combinedgen<-netConstruct(data=ambcomb_class,

data2=sevcomb_class,

measure="sparcc",

dataType="counts",

taxRank = "class",

measurePar = list(rep.num=100),

filtTax="highestVar",

filtTaxPar = list(highestVar=50),

filtSamp="totalReads",

filtSampPar= list(totalReads=500),

normMethod="none",

verbose=3)
